# Levels of high-density lipoprotein cholesterol (HDL-C) among children with steady-state sickle cell disease

**DOI:** 10.1186/1476-511X-9-91

**Published:** 2010-08-27

**Authors:** Magda O Seixas, Larissa C Rocha, Mauricio B Carvalho, Joelma F Menezes, Isa M Lyra, Valma ML Nascimento, Ricardo D Couto, Ájax M Atta, Mitermayer G Reis, Marilda S Goncalves

**Affiliations:** 1 Laboratório de Patologia e Biologia Molecular, Centro de Pesquisa Gonçalo Moniz, Fundação de Pesquisa Oswaldo Cruz (FIOCRUZ), Salvador, Bahia, Brasil; 2 Departamento de Análises Clínicas e Toxicológicas, Faculdade de Farmácia, Universidade Federal da Bahia, Salvador, Bahia, Brasil; 3 Fundação de Hematologia e Hemoterapia do Estado da Bahia (HEMOBA), Salvador, Bahia, Brasil; 4 Hospital Pediátrico Professor Hosannah de Oliveira, Universidade Federal da Bahia, Salvador, Bahia, Brasil

## Abstract

**Background:**

The search for sickle cell disease (SCD) prognosis biomarkers is a challenge. These markers identification can help to establish further therapy, later severe clinical complications and with patients follow-up. We attempted to study a possible involvement of levels of high-density lipoprotein cholesterol (HDL-C) in steady-state children with SCD, once that this lipid marker has been correlated with anti-inflammatory, anti-oxidative, anti-aggregation, anti-coagulant and pro-fibrinolytic activities, important aspects to be considered in sickle cell disease pathogenesis.

**Methods:**

We prospectively analyzed biochemical, inflammatory and hematological biomarkers of 152 steady-state infants with SCD and 132 healthy subjects using immunochemistry, immunoassay and electronic cell counter respectively. Clinical data were collected from patient medical records.

**Results:**

Of the 152 infants investigated had a significant positive association of high-density lipoprotein cholesterol with hemoglobin (P < 0.001), hematocrit (P < 0.001) and total cholesterol (P < 0.001) and a negative significant association with reticulocytes (P = 0.046), leukocytes (P = 0.015), monocytes (P = 0.004) and platelets (P = 0.005), bilirubins [total bilirubin (P < 0.001), direct bilirubin (P < 0.001) and indirect bilirubin (P < 0.001], iron (P < 0.001), aminotransferases [aspartate aminotransferase (P = 0.004), alanine aminotransferase (P = 0.035)], lactate dehydrogenase (P < 0.001), urea (P = 0.030), alpha 1-antitrypsin (P < 0.001), very low-density lipoprotein cholesterol (P = 0.003), triglycerides (P = 0.005) and hemoglobin S (P = 0.002). Low high-density lipoprotein cholesterol concentration was associated with the history of cardiac abnormalities (P = 0.025), pneumonia (P = 0.033) and blood transfusion use (P = 0.025). Lipids and inflammatory markers were associated with the presence of cholelithiasis.

**Conclusions:**

We hypothesize that some SCD patients can have a specific dyslipidemic subphenotype characterized by low HDL-C with hypertriglyceridemia and high VLDL-C in association with other biomarkers, including those related to inflammation. This represents an important step toward a more reliable clinical prognosis. Additional studies are warranted to test this hypothesis and the probably mechanisms involved in this complex network of markers and their role in SCD pathogenesis.

## Background

Sickle cell disease (SCD) clinical outcomes vary widely from mild to severe and the disease has been associated with multi-organ damage and risk of early mortality [1,2]. Acute and chronic clinical manifestations of SCD include vaso-occlusive pain episodes (VOE), impaired blood flow as a result of intravascular sickling in capillaries and small vessels, inflammation processes and high susceptibility to infection. Researchers have found a complex network of associations among laboratory analyses and clinical events predicting a probably risk of death [1,3,4].

The sickle cell disease vaso-occlusive phenomenon has been described as a complex event with the participation of stressed reticulocytes, sickled erythrocytes, leukocytes, platelets and endothelium activation [2,5-8]. Reactive oxygen species (ROS), scavenger molecules and nitric oxide (NO) play important roles as regulators of vascular homeostasis in SCD pathogenesis [9].

Several biomarkers have been associated with SCD clinical prognosis; some, such as fetal hemoglobin (HbF) concentration, leukocytes count and reticulocyte count are considered to be classic [2,5]. Recently, serum lactate dehydrogenase (LDH), a well-known marker of intravascular hemolysis, was described as a biomarker of prognosis in SCD [10]. It has been associated with nitric oxide resistance, priapism, leg ulceration, pulmonary hypertension, and death in SCD patients [11].

We conducted a prospective study to investigate high-density lipoprotein cholesterol (HDL-C) levels, including also determination of total cholesterol, low-density lipoprotein cholesterol (LDL-C), very low-density lipoprotein cholesterol (VLDL-C) and triglycerides to test the hypothesis that they can be used as a marker of prognosis among steady-state sickle cell disease children. This potential biomarker and their association with others laboratory determination and medical history were investigated in order to identify sub-phenotypes associated with the disease.

## Subjects and Methods

### Subjects and Controls

Of 152 steady-state SCD children from Salvador city, state of Bahia, in Brazil were prospectively analyzed for laboratory (biochemical and hematological) markers. Brazil is the largest country in South America, with one of the most heterogeneous populations due to several waves of immigration that have resulted in cultural, socioeconomic, and ethnic diversity in different geographic regions. Salvador is the largest city in Bahia, a Northeastern Brazilian state. Among the local population, 86% is of African origin, and Salvador has the highest incidence of SCD in Brazil [12].

The study was conducted from March 2007 to November 2008 and included patients from the Fundação de Hematologia e Hemoterapia do estado da Bahia (HEMOBA), a reference center attending to sickle cell disease patients who are seen for routine visits at the outpatient clinic. The study also included 132 healthy children randomly selected from the Clinical Laboratory of the Faculdade de Farmácia da Universidade Federal da Bahia (UFBA); these were matched to cases by age, gender and African ethnic origin as a control group. The study was approved by the Fundação de Pesquisa Oswaldo Cruz human subject research board, and all officials responsible provided written informed consent, in accordance with the Declaration of Helsinki of 1975, as revised in 2000.

### Laboratory Methods

Clinical laboratory analyses were performed in the Clinical Analyses Laboratory of the PHAR-UFBA and the Pathology and Molecular Biology Laboratory of the Centro de Pesquisas Goncalo Moniz da Fundação de Pesquisa Oswaldo Cruz. Biochemical markers analyses were measured in serum by immunochemistry assay (A25 system, BIOSYSTEMS SA, Barcelona, Spain). Serum ferritin was measured by immunoassay using an Access^® ^2 Immunoassay system X2 (Beckman Coulter, Fullerton, CA). C-reactive protein (CRP), alpha 1-antitripsin (A1AT) and antistreptolysin-O (ASO) were measured by immunochemistry (Immage^® ^800 system, Beckman Coulter, Fullerton, CA). Hematological analyses were carried out using an electronic cell counter, Coulter Count T-890 (Coulter Corporation, FL, USA). The hemoglobin (Hb) profile and HbF levels were investigated by high performance liquid chromatography (HPLC/VARIANT I; BIO-RAD, CA, USA).

### Definition of Clinical Events

Clinical data were collected from patient medical records. Demographic data were provided by interviews with patients and parents or guardians. Eligibility criteria included only SCD patients of pediatric age. All patients were in the steady-state of the disease when samples were collected; steady-state was characterized as a period without any acute events and no blood transfusion for 120 days prior to blood sampling. Exclusion criteria included infection or inflammatory episodes and previous blood transfusion (within four months prior to the study). To identify possible associations between HDL-C levels and clinical characteristics in SCD we assessed medical history from patients' records, including prevalence of stroke, number of hospitalizations, painful episodes, VOE, infection, pneumonia, priapism, splenomegaly, splenic sequestration, leg ulcers, cardiac abnormalities, respiratory insufficiency and cholelithiasis. Pneumonia was defined as an acute infection of the lung by virus, bacteria or atypical organisms with a clinical outcome that did not meet the criteria for ACS [8].

### Statistical analysis

Baseline characteristics were summarized as means and proportions of selected variables. Distribution of quantitative variables was determined using the Kolmogorov-Smirnov test. Mean values of quantitative variables between groups were compared using the unpaired t-test for normal data distribution and Mann-Whitney for non-normal data. Bivariate correlation analyses were carried out to determine correlations between pairs of variables using Pearson's and Spearman's rank correlation (R). The nonparametric Kruskal-Wallis test was used to compare means among two or more groups as measured by interval variables. The level of 40 mg/dl was considered as a reference range and interactions between low HDL-C (less than 40 mg/dl) and high HDL-C (at least 40 mg/dl) and baseline characteristics were evaluated using independent t-test and Mann-Whitney tests. The interactions between low HDL-C (less than 40 mg/dl) and high HDL-C (at least 40 mg/dl) and specific categorical clinical variables were tested for significance using a χ^2 ^test or Fisher's exact test, taking into account the expected frequency in the cell tables. All tests were considered significant if *p *values were less than .05. Data analyses were performed using Prism 5.01 (Graphpad Software, San Diego, CA), EPIinfo 6.04 (CDC, Atlanta, Georgia) and STATA SE 10 software (StataCorp, Texas, USA).

## Results

First of all we compared the analyses of markers of intravascular hemolysis, hemolysis and hepatic involvement, leukocyte and platelet counts, renal involvement, lipid metabolism, inflammation and Hb profile in order to establish how much are the difference between those markers between control and patients groups (Table 1).

**Table 1:** Patient and control group characteristics

	Patients		Controls		
		
Characteristics	N	Mean ± SD	N	Mean ± SD	*p*
**Age **(Years)	152	9.2 ± 4.0	132	8.7 ± 3.2	
**Gender**					
Male	82	53.9*	68	51.5*	
Female	70	46.1*	64	48.5*	
**Hemoglobins**					
AA	--	---	132	100.0	
SS	103	67.8	--	---	
SC	48	31.5	--	---	
SD	01	0.7	--	---	
**Hemoglobin**					
Fetal **(%)**	142	7.51 ± 6.20	130	0.47 ± 0.46	**<0.001**
**Hemolysis**					
RBC (× 10^6^/cu mm)	152	3.24 ± 0.97	131	4.74 ± 0.39	**<0.001**
Hemoglobin (g/dL)	152	8.93 ± 2.01	131	12.83 ± 1.03	**<0.001**
Hematocrit (%)	152	27.65 ± 6.20	131	38.47 ± 2.78	**<0.001**
Mean Cell Volume (fL)	152	87.44 ± 10.85	131	81.37 ± 5.16	**<0.001**
Mean Cell Hemoglobin (pg)	152	28.29 ± 3.73	131	27.14 ± 1.95	**0.007**
Reticulocyte Count (%)	140	7.61 ± 4.88	122	0.846 ± 0.256	**<0.001**
**Leukocytes**					
Leukocyte Count (× 10^9^/L)	152	13.1 ± 5.8	131	7.0 ± 2.2	**<0.001**
Neutrophil Count (× 10^9^/L)	152	6161.72 ± 3779.49	131	3240.32 ± 1686.15	**<0.001**
Monocyte Count (× 10^9^/L)	152	817.15 ± 481.83	131	488.67 ± 204.90	**<0.001**
**Platelets**					
Platelet Count (× 10^9^/L)	152	403.93 ± 158.66	131	308.21 ± 67.35	**<0.001**
**Lipid metabolism**					
Total Cholesterol (mg/dL)	151	121.12 ± 26.16	124	164.08 ± 34.55	**<0.001**
HDL Cholesterol (mg/dL)	151	35.65 ± 12.34	123	48.90 ± 13.67	**<0.001**
LDL Cholesterol (mg/dL)	151	64.95 ±22.19	123	97.41 ± 33.54	**<0.001**
VLDL Cholesterol (mg/dL)	151	20.44 ± 9.38	123	17.75 ± 10.37	**<0.001**
Triglycerides (mg/dL)	150	102.07 ± 46.86	123	88.31 ± 51.73	**0.002**
**Hemolysis plus Hepatic**					
Aspartate aminotransferase (U/L)	152	48.05 ± 24.92	122	30.28 ± 11.13	**<0.001**
Total bilirubin (mg/dL)	151	2.73 ± 1.76	118	0.49 ± 0.21	**<0.001**
Direct bilirubin (mg/dL)	151	0.66 ± 0.46	118	0.250 ± 0.082	**<0.001**
Indirect bilirubin (mg/dL)	151	2.08 ± 1.59	118	0.244 ± 0.182	**<0.001**
Iron serum (mcg/dL)	126	123.40 ± 119.94	119	71.14 ± 40.31	**<0.001**
Lactate dehydrogenase(U/L)	151	858.22 ± 503.81	119	406.27 ± 132.03	**<0.001**
**Hepatic**					
Alanine aminotransferase (U/L)	152	28.25 ± 21.34	121	17.36 ± 7.10	**<0.001**
Total protein (g/dL)	151	7.33 ± 0.848	119	7.31 ± 0.62	0.695
Albumin (g/dL)	151	4.07 ± 0.675	119	4.24 ± 0.49	0.249
Globulin (g/dL)	151	3.26 ± 0.781	119	3.06 ± 0.63	0.109
Albumin/Globulin ratio	151	1.35 ± .54	112	1.44 ± .42	0.289
**Renal**					
Urea nitrogen (mg/dL)	150	17.73 ± 6.41	120	21.65 ± 5.92	**<0.001**
Creatinine (mg/dL)	151	0.51 ± 0.50	120	0.523 ± 0.185	0.708
**Inflammation**					
C-reactive protein (mg/L)	148	7.08 ± 11.97	102	2.01 ± 2.29	**<0.001**
Alpha 1-antitrypsin (mg/dL)	151	152.50 ± 46.18	129	137.48 ± 43.36	**0.013**
Ferritin (ng/mL)	152	313.32 ± 361.44	117	37.29 ± 28.28	**<0.001**
Antistreptolysin-O(UI/mL)	148	192.70 ± 285.42	101	132.75 ± 131.19	0.181

* percentage Mann-Whitney test

### HDL-C association with markers of hemolysis, inflammation and vascular dysfunction

The high-density lipoprotein cholesterol was positively correlated with red blood cells (RBC), Hb, hematocrit and total cholesterol and urea concentrations and negatively correlated with hematimetric indexes of mean cell volume (MCV), mean cell hemoglobin (MCH) and mean cell hemoglobin concentration (MCHC); reticulocytes, hemoglobin S (HbS), hemolysis and hepatic markers, total leukocytes, monocytes and platelets, alanine aminotransferase (ALT), iron and A1AT. However, it was not correlated with LDL-C. Steady state triglycerides were negatively correlated with RBC, Hb, hematocrit and HDL-C, and positively correlated with HbS, LDH, AST, total bilirubin, platelet, total protein, total cholesterol, and VLDL-C (Table 2).

**Table 2:** Laboratory value associations with HDL-C and Triglycerides in sickle cell disease

	HDL Cholesterol (mg/dL)		Triglycerides (mg/dL)	
	***r***	***p***	***r***	***p***

**Hemoglobin**				
S hemoglobin (%)	-0.311	**0.002**	0.286	**0.005**
Fetal hemoglobin (%)	-0.048	0.644	-0.685	0.685
**Hemolysis**				
RBC (× 10^6^/cu mm)	0.328	**<0.001**	-0.190	**0.019**
Hemoglobin (g/dL)	0.292♣	**<0.001**	-0.202	**0.013**
Hematocrit (%)	0.309	**<0.001**	-0.189	**0.020**
Mean Cell Volume (fL)	-0.273♣	**0.006**	0.126	0.125
Mean Cell Hemoglobin (pg)	-0.284♣	**0.002**	0.111	0.175
Reticulocyte Count (%)	-0.170♣	**0.046**	0.082	0.339
**Leukocyte**				
Leukocyte Count (× 10^9^/L)	-0.198♣	**0.015**	0.081	0.325
Neutrophil Count (× 10^9^/L)	0.017♣	0.838	-0.154	0.061
Monocyte Count (× 10^9^/L)	-0.234	**0.004**	0.139	0.089
**Platelets**				
Platelet Count (× 10^9^/L)	-0.228♣	**0.005**	0.233	**0.004**
**Hemolysis plus Hepatic**				
Aspartate aminotransferase (U/L)	-0.235♣	**0.004**	0.207	**0.011**
Total bilirubin (mg/dL)	-0.298♣	**<0.001**	0.165	**0.044**
Direct bilirubin (mg/dL)	-0.471	**<0.001**	0.035	0.669
Indirect bilirubin (mg/dL)	-0.287	**<0.001**	0.140	0.088
Iron Serum (mcg/dL)	-0.186	**0.038**	0.159	0.076
Lactate dehydrogenase (U/L)	-0.375	**<0.001**	0.167	**0.041**
**Hepatic**				
Alanine aminotransferase (U/L)	-0.172	**0.035**	0.075	0.364
Total protein (g/dL)	-0.021♣	0.793	0.274	**0.001**
Albumin (g/dL)	0.102	0.213	0.142	0.083
Globulin (g/dL)	-0.124♣	0.129	0.133	0.104
Albumin/Globulin ratio	0.033	0.689	-0.033	0.684
**Renal**				
Urea nitrogen (mg/dL)	0.178	**0.030**	0.020	0.806
Creatinine (mg/dL)	0.118	0.152	0.105	0.201
**Lipid metabolism**				
Total Cholesterol (mg/dL)	0.299♣	**<0.001**	0.268	**0.001**
HDL Cholesterol (mg/dL)	----	----	-0.228	**0.005**
LDL Cholesterol (mg/dL)	-0.083♣	0.312	0.068	0.409
VLDL Cholesterol (mg/dL)	-0.242	**0.003**	0.998	**<0.001**
Triglycerides (mg/dL)	-0.228	**0.005**	----	----
**Inflammation**				
C-reactive protein (mg/L)	0.048	0.563	-0.031	0.714
Alpha 1 antitrypsin (mg/dL)	-0.327♣	**<0.001**	-0.074	0.378
Ferritin (ng/mL)	-0.032	0.699	0.102	0.220
Antistreptolysin O (UI/mL)	-0.079	0.339	0.157	0.058

Spearman or Pearson correlation coefficients (r) and p values (*p*) ♣ r = Pearson correlation coefficient

We next determined whether the levels of HDL-C in SCD group (HDL-C less than 40 mg/dl vs. 40 mg/dl or more) showed difference among the laboratorial markers. In the first group, there were 80 *HBSS *and 23 *HBSC *patients, and in the second group, there were 23 HBSS and 25 HBSC patients. Sickle cell patients with low HDL-C presented lower RBC counts as well as Hb and hematocrit concentrations than patients from the group with normal HDL-C levels. The low concentration HDL-C group had higher erythroblast, leukocyte, platelet, neutrophil, monocyte and reticulocyte counts and higher iron, AST, total bilirubin, direct bilirubin, indirect bilirubin, LDH and A1AT concentrations. There was no difference in LDL-C concentration between the two HDL-C subgroups, but the VLDL-C and triglycerides concentrations were higher in the low HDL-C group (Table 3).

**Table 3:** Laboratory values for sickle cell disease patients with different steady-state levels of HDL-C

	**HDL less than 40 mg/dL		***HDL at least 40 mg/dL		
		
	**N**	**Mean ± SD**	**N**	**Mean ± SD**	*p
	
**Hemolysis**					
RBC (× 10^6^/cu mm L)	103	3.01 ± 0.85	48	3.75 ± 1.0	**<0.001**
Hemoglobin (g/dL)	103	8.57 ± 2.02	48	9.76 ± 1.75	**0.001**
Hematocrit (%)	103	26.42 ± 6.17	48	30.43 ± 5.37	**<0.001**
Mean Cell Volume (fL)	103	89.17 ± 10.34	48	83.52 ± 11.01	**0.003**
Mean Cell Hemoglobin (pg)	103	28.94 ± 3.52	48	26.82 ±3.79	**0.001**
Mean Cell Hemoglobin Concentration (%)	103	32.44 ± 0.96	48	32.07 ± 0.86	**0.025**
Erythroblast (%)	103	1.90 ± 2.31	48	1.02 ± 2.48	**0.034**
Reticulocyte count (%)	97	8.34 ± 4.55	42	5.90 ± 5.25	**0.006**
**Hemoglobins**					
S hemoglobin (%)	97	79.22 ± 16.16	44	60.75 ± 18.22	**<0.001**
Fetal hemoglobin (%)	97	7.55 ± 5.99	44	7.41 ± 6.78	0.899
**Leukocyte**					
Leukocyte Count (× 10^9^/L)	103	14105.83 ± 6085.37	48	10868.75 ± 4416.13	**0.001**
Neutrophil Count (× 10^9^/L)	103	6723.36 ± 4167.75	48	4971.83 ± 2459.10	**0.002**
Monocyte Count (× 10^9^/L)	103	910.35 ± 499.84	48	604.69 ± 361.83	**0.004**
**Platelets**					
Platelet Count (× 10^9^/L)	103	424.90 ± 160.26	48	357.69 ± 148.02	**0.015**
**Hemolysis plus Hepatic**					
Aspartate aminotransferase (U/L)	103	51.45 ± 26.29	48	40.65 ± 20.32	**0.013**
Total bilirubin (mg/dL)	103	3.13 ± 1.82	48	1.88 ± 1.25	**<0.001**
Direct bilirubin (mg/dL)	103	0.79 ± 0.47	48	0.38 ± 0.25	**<0.001**
Indirect bilirubin (mg/dL)	103	2.34 ± 1.68	48	1.50 ± 1.20	**0.002**
Iron serum (mcg/dL)	95	136.65 ± 133.77	31	82.77 ± 40.17	**0.029**
Lactate dehydrogenase (U/L)	103	977.19 ± 524.50	48	602.92 ± 339.59	**<0.001**
**Lipid metabolism**					
Total Cholesterol (mg/dL)	103	116.49 ± 25.17	48	131.06 ± 25.73	**0.001**
LDL Cholesterol (mg/dL)	103	65.78 ± 21.47	48	63.19 ± 23.81	0.506
VLDL Cholesterol (mg/dL)	103	21.62 ± 10.31	48	17.90 ± 6.38	**0.023**
Triglycerides (mg/dL)	102	107.74 ± 51.64	48	90.02 ± 31.86	**0.030**
**Hepatic**					
Alanine aminotransferase (U/L)	103	29.90 ± 22.10	48	24.58 ± 19.56	0.156
Total protein (g/dL)	103	7.40 ± .89	48	7.46 ± .82	0.684
Albumin (g/dL)	103	4.01 ± .75	48	4.23 ± .59	0.054
Globulin (g/dL)	103	3.36 ± .85	48	3.23 ± .72	0.333
Albumin/Globulin ratio	103	1.34 ± .55	48	1.38 ± .53	0.709
**Renal**					
Urea nitrogen (mg/dL)	102	17.25 ± 6.70	47	18.77 ± 5.73	0.181
Creatinine (mg/dL)	103	0.49 ±0.50	47	0.57 ± 0.50	0.315
**Inflammation**					
C-reactive protein (mg/L)	101	7.79 ± 14.02	46	5.39 ± 5.18	0.133
Alpha 1 antitrypsin (mg/dL)	102	163.13 ± 44.06	48	128.92 ± 42.13	**<0.001**
Ferritin (ng/mL)	103	300.76 ± 399.59	46	323.46 ± 348.87	0.740
Antistreptolysin O (UI/mL)	101	198.62 ± 288.98	46	183 ± 282.56	0.759

* unpaired t-test ** 80 HBSS and 23 HBSC ***23 HBSS and 25 HBSC

### Association of HDL-C with sickle cell disease clinical history

We assessed possible associations between HDL-C levels and a series of clinical characteristics in SCD medical history, including prevalence of stroke, number of hospitalizations, painful episodes, VOE, infection, pneumonia, priapism, splenomegaly, splenic sequestration, leg ulcers, cardiac abnormalities, respiratory insufficiency and cholelithiasis. To compare these categorical variables with HDL-C concentration, we divided patients into two groups. The low HDL-C group (less than 40 mg/dl) comprised 103 sickle cell disease patients (80 HBSS and 23 HBSC), with an HDL-C range of 16-39 mg/dl and mean of 28.95 mg/dl. The high HDL-C group (at least 40 mg/dl) comprised 48 SCD patients (23 HBSS and 25 HBSC), with an HDL-C range of 41-85 mg/dl and mean of 51.2 mg/dl.

The prevalence of pneumonia (OR = 2.42, 95%CI: 1.06-5.53; P = 0.033) and the prevalence of cardiac abnormalities (OR = 2.88, 95%CI: 1.12-7.59, P = 0.025) were significantly different between the HDL-C groups. Forty-one children in the low HDL-C group had cardiac abnormalities typical of hemolytic anemia on auscultation. However, among these, 24 had electrocardiograph arrhythmia, and 3 had tricuspid regurgitant jet velocity of at least 2.6 m/sec, indicating a possible presence of pulmonary hypertension. These results were obtained from previously performed echocardiograms that were not preformed at the same time of the present study. The low HDL-C concentration group underwent more blood transfusions (OR = 2.52, 95%CI: 1.11-5.77, P = 0.025).

**Figure 1:**
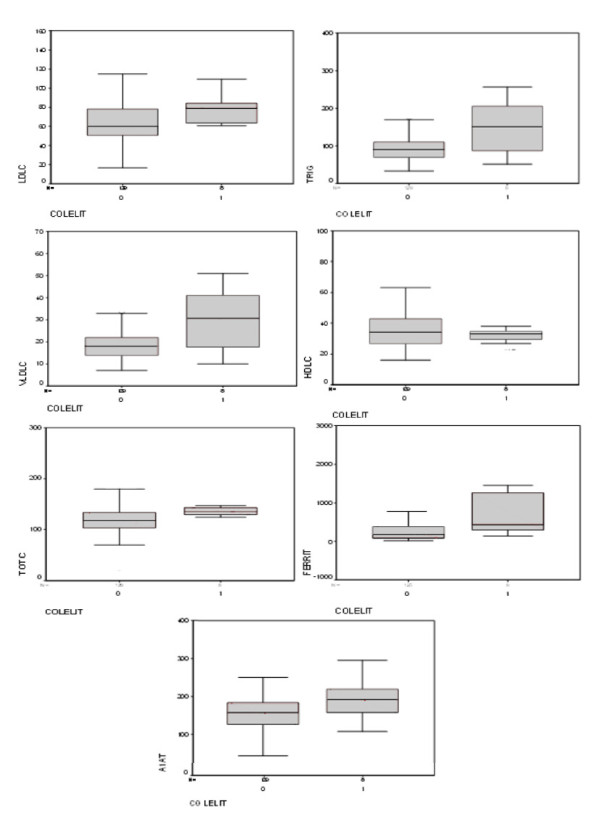
**Box-plots of cholelithiasis and biochemical markers among sickle cell disease steady-state children**. Number 0 represents absence and 1 presence of cholelithiasis (COLELIT) analyzed accord to different serum concentrations of triglycerides (TRIG) (P = 0.047), very-low density lipoprotein cholesterol (VLDLC) (P = 0.044), low-density lipoprotein cholesterol (LDLC) (P = 0.033), total cholesterol (P = 0.007), high-density lipoprotein cholesterol (HDLC) (P = 0.349), alpha 1-antitrypsin (A1AT) (P = 0.040) and ferritin (FERRIT) (P = 0.008). The p values were estimated by the Kruskal-Wallis test.

High serum levels of LDL-C, VLDL-C and total cholesterol lipoproteins, TRIG, ferritin and A1AT but not HDL-C were associated with the occurrence of cholelithiasis (Figure 1).

## Discussion

The present study analyzed levels of HDL-C in steady-state children with SCD. Children with SCD, even in steady-state, have differences in several biomarkers as compared to healthy age-matched children [13]. Those differences are related to numerous mechanisms associated with infection, inflammation and VOE in the disease [1,2]. Several biomarkers associated with hemolysis, inflammation, renal metabolism, hepatic metabolism, and lipid metabolism in children with SCD and healthy subjects were studied, and the findings of normal concentrations of protein and globulin as well as the albumin/globulin ratio among the SCD patients suggest an absence of early severe liver cell damage in the studied group [13]. Normal levels of creatinine in the patient group confirm previous observations that an increased rate of creatinine secretion by dysfunctional renal tubules may lead to a falsely normal plasma creatinine and creatinine clearance. A more accurate evaluation of different aspects of SCD nephropathy, emphasizing proteinuria and hyperfiltration, needs to be developed in children in order to detect early renal alteration [14-16].

Hypocholesterolemia has been described in SCD patients with significantly decreased LDL-C and HDL-C [17-22] and has been also described for our group as a potential biomarker for SCD clinical severity [23]. A negative association was found for HDL-C and VLDL-C, which was directly associated with triglycerides. Triglyceride-rich VLDL-C particles availability may play an important role in lipid oxidization in SCD patients. It has been suggested that VLDL-C is an important factor for atherosclerosis development. VLDL-C particles assemble by a complex process that includes an apolipoprotein B (apoB)-containing VLDL precursor and a VLDL-sized lipid droplet lacking apoB. Both particles fuse to produce a mature VLDL particle [24]. The increase of triglycerides probably contributes to an increase in the hepatic production of VLDL-C, increasing the number of receptors for LDL-C that is extensively metabolized, decreasing its serum levels. However, the role of cholesterol and triglycerides and the regulation of assembly and production of VLDL-C are poorly understood.

A negative association was observed between LDH and HDL-C, showing that HDL-C, as measured by its concentration, may function as a prognostic marker of intravascular hemolysis and endothelial dysfunction given its anti-inflammatory, anti-oxidative, anti-aggregation, anti-coagulant and pro-fibrinolytic activities [25,26].

Sickle cell disease patients with higher HDL-C levels presented a low risk of hemolysis and endothelial dysfunction, including lower reticulocyte and erythroblast counts as well as a lower HbS concentration and it may be related to the high consumption of cholesterol due to acceleration of blood marrow cell production during hemolysis crisis. Sickle cell disease patients with higher HDL-C levels had lower leukocyte, monocyte and platelet counts as well as a lower concentration of hepatic and hemolytic markers and significantly lower VLDL-C, triglycerides and A1AT concentrations; this may reflect the action of the anti-inflammatory and anti-oxidative properties of this biomarker [25,26]. The high-density lipoprotein cholesterol removes excess cholesterol from peripheral tissues and transports it to the liver for excretion via bile by reverse cholesterol transport. The high-density lipoprotein is made up of several particles with different composition and function [24,27,28].

Further confirmation of these associations came from comparing HDL-C concentrations and patients' clinical records, which revealed a higher occurrence of pneumonia and cardiac abnormalities among those with lower HDL-C levels. The results related to pneumonia risk can be explained by the production of auto-antibodies specific to oxidized phospholipids; these auto-antibodies have been shown to inhibit macrophage uptake of oxidized LDL and to provide protection against virulent pneumococcal infection [29]. Low levels of HDL-C are an important cardiovascular risk factor, and HDL-C and apoA-I have been shown to decrease lesions and improve vascular reactivity in animal models of atherosclerosis and in humans; these changes may be due to the reduction of oxidized lipids and the enhancement of reverse cholesterol transport [30]. The presence of pulmonary hypertension was shown to be associated with several laboratory test alterations [31]. Recent study has also demonstrated the important role of the apolipoprotein pathway and its association with endothelial dysfunction in SCD patients with pulmonary hypertension [31].

Patients with lower HDL-C levels were also likely to have had more blood transfusions; this can be linked with a more severe clinical course of disease, once that it is a therapeutic strategy used to prevent several clinical symptoms, such as stroke [1].

It is well known that gallstones in patients with hemolytic anemia are said to be calcium bilirubinate stones. In view our results of correlation of cholesterol and triglycerides with hemolysis, we propose that the stones in SCD patients could be related directly to hemolysis and bilirubin generation, and indirectly to cholesterol and lipids and it could be a novel observation and needs to be confirmed by further studies. The association of acute-phase proteins and cholelithiasis may be explained by the response to stress due to traumatic injury or infection-related mechanisms including hypermetabolism and protein catabolism associated with a cytokine-driven inflammatory response.

## Conclusions

In conclusion, we hypothesize that some SCD patients can have a specific dyslipidemic subphenotype characterized by low HDL-C with hypertriglyceridemia and high VLDL-C in association with other biomarkers, including those related to inflammation. This represents an important step toward a more reliable clinical prognosis. Additional studies are warranted to test this hypothesis and the probably mechanisms involved in this complex network of markers and their role in SCD pathogenesis.

## Abbreviations

SCD: Sickle cell disease; VOE: vaso-occlusive pain episodes; HDL-C: high-density lipoprotein cholesterol; LDL-C: low-density lipoprotein cholesterol; VLDL-C: very low-density lipoprotein cholesterol; LDH: lactate dehydrogenase; CRP: C-reactive protein; A1AT: alpha 1-antitripsin; ASO: antistreptolysin-O; HB: hemoglobin; HBF: hemoglobin fetal; HPLC: high performance liquid chromatography; RBC: red blood cells; MCV: mean cell volume; MCH: mean cell hemoglobin; MCHC: mean cell hemoglobin concentration; HBS: hemoglobin S; COLELIT: cholelithiasis.

## Competing interests

The authors declare that they have no competing interests.

## Authors' contributions

MOS performed experiments and analyzed the results; LR, MBC, JM, RDC, and AMA performed experiments; IML, VMLN and LR performed clinical evaluation of patients; MGR analyzed the results; and MSG was the principal investigator and takes primary responsibility for the paper, designed the research, analyzed the results and wrote the paper. All authors read and approved the final manuscript.
